# Higher postoperative complication rates in patients with systemic lupus erythematosus undergoing surgical and obstetric procedures: a nationwide retrospective matched cohort study

**DOI:** 10.1007/s00296-026-06316-9

**Published:** 2026-10-09

**Authors:** Francisco Eduardo Silva, Mirhelen Mendes de Abreu

**Affiliations:** 1 Universidade Federal do Rio de Janeiro, Campus Macaé, Rua Aluízio da Silva Gomes, 50. Granja dos Cavaleiros. Macaé, Rio de Janeiro, RJ 27930-560 Brazil; 2 Universidade Federal do Rio de Janeiro, Campus Cidade Universitária, Rua Professor Rodolpho Paulo Rocco, 255, Rio de Janeiro, RJ 21941-913 Brazil

**Keywords:** Lupus erythematosus, systemic, Perioperative care, Postoperative complications, Immunosuppressive agents, Obstetric surgical procedures, Surgical procedures, operative

## Abstract

Population-level evidence on postoperative outcomes in systemic lupus erythematosus (SLE) remains limited. To compare postoperative outcomes between patients with SLE and matched controls and identify factors associated with complications and algorithm-defined disease reactivation. We conducted a nationwide retrospective matched cohort study using DATASUS data. Patients with SLE were matched 1:5 to controls by sex, age group, geographic macro-region, preoperative surgical risk, and surgical category. Postoperative outcomes were assessed over 180 days. Multivariable analyses assessed factors associated with outcomes within the SLE cohort. Sensitivity analyses comprised restriction to elective procedures and exclusion of patients classified with cutaneous lupus. The cohort included 1,708 patients with SLE and 8,540 controls. SLE was associated with increased risks of surgical (RR 2.20, 95% CI 1.91–2.54), clinical (RR 3.30, 95% CI 2.53–4.30), infectious (RR 3.66, 95% CI 2.88–4.64), and other procedure- or care-related complications (RR 11.33, 95% CI 6.19–20.76). Among patients undergoing obstetric procedures, SLE was associated with obstetric complications (RR 1.87, 95% CI 1.37–2.54). Within the SLE cohort, cardiovascular risk was independently associated with higher odds of clinical (adjusted OR 2.75, 95% CI 1.36–5.54) and other procedure- or care-related complications (adjusted OR 5.46, 95% CI 1.84–16.18). Associations between SLE and surgical, clinical, and infectious complications remained significant in both sensitivity analyses. Algorithm-defined SLE reactivation occurred in 3.3%. Patients with SLE had higher risks of several postoperative complications than matched controls. The contributions of disease activity and immunosuppressive treatment warrant further investigation.

**Supplementary Information:**

The online version contains supplementary material available at https://doi.org/10.1007/s00296-026-06316-9.

## Introduction

Systemic lupus erythematosus (SLE) is a multisystem autoimmune disease characterized by heterogeneous clinical manifestations influenced by genetic, immunologic, and environmental factors. This heterogeneity poses ongoing challenges for clinical management and the development of robust evidence [1]. 

Surgical trauma triggers a systemic inflammatory response that is typically adaptive in immunocompetent individuals. In patients with SLE, this response interacts with preexisting immune dysregulation and the effects of chronic immunosuppressive therapy, potentially magnifying perioperative vulnerability. This interaction remains poorly characterized in scientific literature [2–5]. 

Evidence regarding surgical and obstetric outcomes in SLE remains limited, particularly at the population level [2–5]. Although previous studies have suggested increased postoperative morbidity, the pattern of risk across different surgical and obstetric settings remains incompletely characterized. Lupus-specific perioperative risk is also not explicitly addressed in major perioperative and anesthetic guidelines.

The primary objective was to compare postoperative outcomes between patients with SLE undergoing abdominal, pelvic, urologic, or obstetric procedures and matched controls without autoimmune disease using population-based data from DATASUS, the national administrative database of Brazil’s Unified Health System. As a secondary objective, we evaluated whether cardiovascular risk and individual SLE clinical subtypes were associated with postoperative complications and algorithm-defined disease reactivation within the SLE cohort.

## Methods

### Study design and data sources

We conducted a nationwide retrospective cohort study using administrative health data from the Brazilian Unified Health System (Sistema Único de Saúde [SUS]) to evaluate postoperative outcomes among patients with SLE undergoing abdominal, pelvic, urologic, or obstetric procedures between 2008 and 2018 (Figure S1. Online Resource). This study was reported in accordance with the Strengthening the Reporting of Observational Studies in Epidemiology (STROBE) statement for cohort studies.

Data were obtained from the Department of Informatics of SUS (DATASUS) (See Online Resource, Tables S1 to S6). Three administrative databases were integrated: the Hospital Admission Authorization database (Autorização de Internação Hospitalar [AIH]), the Individualized Outpatient Production Bulletin (Boletim de Produção Ambulatorial Individualizado [BPA-I]), and the Authorization for High-Complexity Outpatient Procedures database (Autorização de Procedimentos de Alta Complexidade [APAC]). Together, these databases provide complementary information on inpatient admissions, outpatient procedures, diagnoses, and high-complexity treatments within SUS [6, 7]. 

Records were linked through a unique anonymised patient identifier, allowing longitudinal reconstruction of healthcare trajectories across the three databases. Linkage consistency was assessed through cross-validation, duplicate removal, and predefined checks of temporal consistency. Detailed information on database architecture, variable definitions, coding strategies, and data-quality procedures is provided in the Online Resource (Tables S1 to S6).

### Identification of patients with SLE

Patients were identified using a predefined algorithm combining diagnostic and treatment records. Eligible individuals were required to have ICD-10 codes compatible with SLE or lupus-related disease (M32.x, L93.x, or N08.x), together with outpatient treatment records compatible with SLE management according to the Brazilian Clinical Protocol and Therapeutic Guidelines in effect during the study period. Patients were additionally required to have undergone an abdominal, pelvic, urologic, or obstetric procedure recorded in the AIH database and to have documented use of immunosuppressive treatment during the 12 months preceding surgery [6–11].

*Clinical subtype classification.* Patients were classified into three mutually exclusive administrative clinical subtypes according to ICD-10 coding: multisystemic SLE (M32.x), cutaneous lupus (L93.x), and lupus nephritis (N08.x). Classification required at least two records of the same subtype-specific ICD-10 code within a 6-month window before or after the index procedure, together with at least two corresponding procedure records defined by the 2013 Brazilian Clinical Protocol and Therapeutic Guidelines for systemic lupus erythematosus, which was in effect during the study period. Detailed diagnostic definitions and coding algorithms are provided in the Online Resource.

### Control group and matching

The control group consisted of individuals undergoing the same categories of abdominal, pelvic, urologic, or obstetric procedures who had no recorded diagnosis of SLE or other autoimmune disease and no condition associated with secondary immunosuppression. Individuals with malignant neoplasms, other autoimmune diseases requiring immunosuppressive therapy, AIDS, or other conditions associated with secondary immunosuppression were excluded.

Each patient with SLE was matched to five controls in a 1:5 ratio according to sex, age group, geographic macro-region, and preoperative surgical risk category. When more than five eligible controls were available within the same matching stratum, the first five eligible records were selected according to record availability. Matching accounted for surgical category, but not for the exact individual procedure.

Post-matching covariate balance was assessed using standardised mean differences (SMDs), with values below 0.10 considered indicative of adequate balance. All reported baseline variables met this criterion in the final matched cohort.

### Sample size

No a priori sample size calculation was performed because this was a retrospective population-based study including all eligible patients with SLE identified in the DATASUS databases during the predefined study period. The final SLE cohort comprised 1,708 patients. A 1:5 matching ratio was used to increase statistical efficiency while retaining all eligible cases, resulting in 8,540 matched controls and a total analytical sample of 10,248 individuals.

### Surgical procedures and preoperative risk

Surgical procedures were identified using SUS procedure codes recorded in the AIH database and classified into four predefined categories: elective surgery, emergency inflammatory surgery, abdominal trauma surgery, and obstetric procedures.

Preoperative clinical characteristics and risk domains were operationalized using diagnostic and procedure codes available in the administrative databases, based on domains derived from the 3rd Brazilian Guideline for Perioperative Cardiovascular Evaluation [10]. The variables included cardiovascular risk, clinical comorbidity, multimorbidity, and obstetric risk. Detailed operational definitions and corresponding ICD-10 and SUS procedure codes are provided in the Online Resource. The index procedure was defined as the eligible abdominal, pelvic, urologic, or obstetric procedure used to establish cohort entry [10, 11].

### Postoperative outcomes

Postoperative complications were identified using diagnostic and procedure records during the 180 days following the index procedure. Outcomes included surgical, clinical, infectious, obstetric, and other procedure- or care-related complications, as well as algorithm-defined SLE reactivation. Outcome categories were analysed independently, allowing patients to contribute to more than one complication category when clinically plausible.

Five categories of postoperative complications were evaluated: (1) surgical complications, including reoperation, abscess, wound dehiscence, and hemorrhagic events; (2) clinical complications, including organ failure and cardiovascular or respiratory events; (3) infectious complications, including sepsis and surgical-site, urinary, or respiratory infections; (4) obstetric complications, including miscarriage, hemorrhage, eclampsia, and preeclampsia; and (5) other procedure- or care-related complications not classified within the preceding categories. The latter category was identified using ICD-10 codes related to complications of medical or surgical procedures and care, including T81.x, T88.x, Y60.x, Y61, Y65.2, Y83, and K91.4.

Postoperative SLE reactivation was defined using an administrative algorithm based on a new record compatible with active SLE together with resumption or escalation of immunosuppressive treatment. Because direct clinical measures of disease activity were unavailable, this outcome was considered an administrative proxy and not a clinically adjudicated flare.

### Statistical analysis

Continuous variables were summarized as means and standard deviations, and categorical variables as absolute and relative frequencies. Categorical variables were compared using Pearson’s chi-square test or Fisher’s exact test, as appropriate. Continuous variables were compared using analysis of variance when applicable.

Postoperative complication frequencies were compared between patients with SLE and matched controls. Risk ratios (RRs) and corresponding 95% confidence intervals (CIs) were estimated by comparing the occurrence of surgical, clinical, infectious, obstetric, and other procedure- or care-related complications between patients with SLE and matched controls during the 180-day postoperative follow-up. Descriptive and comparative analyses of obstetric complications were restricted to patients undergoing obstetric procedures.

Separate multivariable logistic regression models were used to evaluate associations between cardiovascular risk and postoperative outcomes and between each SLE clinical subtype and postoperative outcomes. Each clinical subtype was modeled separately as a binary variable. For obstetric complications, multivariable logistic regression analyses were restricted to patients undergoing an obstetric index procedure. Models were adjusted for age, sex, and multimorbidity. Results are reported as adjusted odds ratios (ORs) with 95% confidence intervals (CIs).

To assess the potential influence of surgical urgency, a sensitivity analysis restricted the cohort to elective procedures. Because administrative coding may not reliably distinguish severe or refractory cutaneous lupus from incompletely coded systemic SLE, the second sensitivity analysis excluded patients classified with cutaneous lupus (L93). In both sensitivity analyses, adjusted risk ratios and corresponding 95% confidence intervals were estimated, with covariate adjustment as specified in the note to Table 6. All analyses were considered exploratory, and no adjustment was made for multiple comparisons. No missing data were present for the variables included in the final analytic dataset.

### Ethical considerations

The study was approved by the Research Ethics Committee of Hospital Universitário Clementino Fraga Filho on March 20, 2019 (CAAE 23962819.8.0000.5257). Data were obtained through an official request to DATASUS under protocol 25000.134828/2015-86 and analysed using anonymised patient identifiers. The requirement for informed consent was waived because the study used retrospective, anonymized administrative data.

ChatGPT (GPT-5.6 Sol, OpenAI) was used in August 2026 during manuscript revision to assist with language editing, text organisation, and refinement of author-generated text. All AI-assisted output was critically reviewed, verified, and edited by the authors. Artificial intelligence was not used to generate or analyze study data, perform statistical analyses, independently interpret results, or select scientific references. All scientific content and final editorial decisions remained the sole responsibility of the authors.

## Results

A total of 10,248 individuals were included in the analysis, comprising 1,708 patients with SLE and 8,540 matched controls. After 1:5 matching, the groups were well balanced across demographic, geographic, clinical, and surgical characteristics, with standardised mean differences below 0.10 for all reported variables (Table 1). Women represented 92.1% of the SLE group and 92.8% of controls. Clinical comorbidity was present in 21.6% of both groups, while multimorbidity was identified in 33.5% of each group. Cardiovascular risk was present in 7.4% of patients with SLE and 6.4% of controls, and obstetric risk in 10.1% and 11.5%, respectively. Surgical categories were similarly distributed between groups, with obstetric procedures being the most frequent (39.8% vs. 40.8%), followed by elective surgery (33.6% vs. 34.0%) and emergency inflammatory surgery (25.9% vs. 24.6%).

**Table 1:** Baseline characteristics of patients with systemic lupus erythematosus and matched controls

Variable	SLE (*n* = 1,708)	Controls (*n* = 8,540)	p-value	SMD
Age range, n (%)			0.999	0.01
16–20 years	161 (9.4)	781 (9.1)		
21–25 years	308 (18.0)	1,492 (17.4)		
26–30 years	309 (18.1)	1,556 (18.2)		
31–35 years	292 (17.1)	1,482 (17.5)		
36–40 years	200 (11.7)	1,012 (11.9)		
41–45 years	147 (8.6)	756 (8.9)		
46–50 years	113 (6.6)	549 (6.4)		
51–55 years	67 (3.9)	340 (4.0)		
>55 years	111 (6.5)	572 (6.7)		
Female sex, n (%)	1,573 (92.1)	7,921 (92.8)	0.370	0.03
Geographic region, n (%)			0.954	0.02
Central-West	135 (7.9)	684 (8.0)		
Northeast	371 (21.7)	1,779 (20.8)		
North	92 (5.4)	464 (5.4)		
Southeast	889 (52.0)	4,495 (52.6)		
South	221 (12.9)	1,118 (13.1)		
Clinical comorbidity, n (%)	369 (21.6)	1,844 (21.6)	1.000	0.00
Multimorbidity, n (%)	573 (33.5)	2,859 (33.5)	0.978	0.00
Cardiovascular risk, n (%)	126 (7.4)	545 (6.4)	0.143	0.04
Obstetric risk, n (%)	172 (10.1)	982 (11.5)	0.096	0.05
Surgical category, n (%)			0.602	0.03
Elective surgery	574 (33.6)	2,903 (34.0)		
Emergency inflammatory surgery	443 (25.9)	2,099 (24.6)		
Abdominal trauma surgery	12 (0.7)	50 (0.6)		
Obstetric procedures	679 (39.8)	3,488 (40.8)		

Patients with SLE were matched 1:5 to controls according to sex, age group, geographic macro-region, preoperative surgical risk category, and surgical category. Standardised mean differences (SMDs) < 0.10 indicate adequate covariate balance

*SLE* Systemic lupus erythematosus, *SMD* Standardised mean difference

Among patients with SLE, clinical characteristics differed according to disease subtype (Table 2). Women accounted for 96.3% of patients with multisystemic SLE, 93.5% of those with cutaneous lupus, and 68.8% of those with lupus nephritis (*p* < 0.001). Clinical comorbidity was more frequent among patients with lupus nephritis (47.5%) than among those with multisystemic SLE (17.5%) or cutaneous lupus (19.1%; *p* < 0.001). Cardiovascular risk was also more frequent in patients with lupus nephritis (12.4%) than in those with multisystemic SLE (6.3%) or cutaneous lupus (7.4%; *p* = 0.011), whereas multimorbidity did not differ significantly across subtypes (32.7%, 35.2%, and 32.7%, respectively; *p* = 0.591).

**Table 2:** Clinical, obstetric, and surgical characteristics of patients with SLE according to clinical subtype

Variable	Multisystemic SLE (*n* = 935)	Cutaneous lupus (*n* = 571)	Lupus nephritis (*n* = 202)	p-value
Female sex, n (%)	900 (96.3)	534 (93.5)	139 (68.8)	< 0.001
Clinical comorbidity, n (%)	164 (17.5)	109 (19.1)	96 (47.5)	< 0.001
Multimorbidity, n (%)	306 (32.7)	201 (35.2)	66 (32.7)	0.591
Cardiovascular risk, n (%)	59 (6.3)	42 (7.4)	25 (12.4)	0.011
Obstetric risk, n (%)	106 (11.3)	58 (10.2)	8 (4.0)	0.007
Surgical category, n (%)				0.039
Elective surgery	316 (33.8)	191 (33.5)	67 (33.2)	
Emergency inflammatory surgery	228 (24.4)	143 (25.0)	72 (35.6)	
Abdominal trauma surgery	7 (0.7)	4 (0.7)	1 (0.5)	
Obstetric procedures	384 (41.1)	233 (40.8)	62 (30.7)	

Patients with SLE were classified into mutually exclusive administrative clinical subtypes using predefined diagnostic and treatment criteria. Comparisons were performed using the chi-square test for categorical variables. Abbreviation: SLE, systemic lupus erythematosus

The distribution of surgical procedures differed across SLE subtypes (*p* = 0.039). Emergency inflammatory procedures were more frequent among patients with lupus nephritis (35.6%) than among those with multisystemic SLE (24.4%) or cutaneous lupus (25.0%), whereas obstetric procedures were less frequent in the nephritis subgroup (30.7% vs. 41.1% and 40.8%, respectively).

During the 180-day follow-up after the index procedure, patients with SLE had higher frequencies of all evaluated categories of postoperative complications than matched controls (Table 3). Surgical complications occurred in 14.2% of patients with SLE and 6.4% of controls (RR 2.20, 95% CI 1.91–2.54; *p* < 0.001). Clinical complications occurred in 5.1% and 1.5%, respectively (RR 3.30, 95% CI 2.53–4.30; *p* < 0.001), and infectious complications in 6.6% and 1.8% (RR 3.66, 95% CI 2.88–4.64; *p* < 0.001). Among patients who underwent obstetric procedures, obstetric complications occurred in 52 of 679 patients with SLE (7.7%) and 143 of 3,488 matched controls (4.1%), corresponding to a crude RR 1.87, 95% CI 1.37–2.54; *p* < 0.001). Other procedure- or care-related complications showed the largest relative difference, occurring in 2.0% of patients with SLE and 0.2% of controls (RR 11.33, 95% CI 6.19–20.76; *p* < 0.001). Algorithm-defined SLE reactivation was identified in 56 patients (3.3%).

**Table 3:** Postoperative complications during the 180-day follow-up in patients With SLE and matched controls

Outcome	SLE (*n* = 1,708)	Controls (*n* = 8,540)	RR (95% CI)	p-value
Surgical complications	242 (14.2)	550 (6.4)	2.20 (1.91–2.54)	< 0.001
Clinical complications	87 (5.1)	132 (1.5)	3.30 (2.53–4.30)	< 0.001
Infectious complications	112 (6.6)	153 (1.8)	3.66 (2.88–4.64)	< 0.001
Obstetric complications**	52 (7.7)	143 (4.1)	1.87 (1.37–2.54)	< 0.001
Other procedure- or care-related complications*	34 (2.0)	15 (0.2)	11.33 (6.19–20.76)	< 0.001
Algorithm-defined SLE reactivation	56 (3.3)	—	—	—

Postoperative complications were recorded during the 180-day follow-up after the index procedure. Risk ratios (RRs) with 95% confidence intervals (CIs) compare postoperative complication frequencies between patients with SLE and matched controls. Algorithm-defined SLE reactivation was assessed only within the SLE cohort. Percentages for obstetric complications were calculated among patients undergoing obstetric procedures (SLE *n* = 679; controls *n* = 3,488); percentages for the other postoperative complication categories were calculated using the total matched-cohort denominators

*Other procedure- or care-related complications comprised events related to medical or surgical procedures or care that were not classified within the preceding complication categories

**Analyses of obstetric complications were restricted to patients undergoing an obstetric index procedure

Patients with SLE had a longer mean hospital stay than matched controls (4.92 vs. 3.08 days, *p* < 0.001) and were more frequently admitted to the ICU (8.7% vs. 3.4%, *p* < 0.001) (Table 4). Mean ICU length of stay did not differ significantly between groups (4.99 vs. 4.13 days, *p* = 0.133). In-hospital mortality was higher among patients with SLE than among matched controls (4.4% vs. 0.8%, *p* < 0.001).

**Table 4:** Hospital outcomes and intensive care utilization in patients with systemic lupus erythematosus and matched controls

Outcome	SLE (*n* = 1,708)	Controls (*n* = 8,540)	p-value
Hospital length of stay, mean (SD), days	4.92 (6.45)	3.08 (7.20)	< 0.001
ICU admission, n (%)	149 (8.7)	290 (3.4)	< 0.001
ICU length of stay, mean (SD), days	4.99 (2.60)	4.13 (7.22)	0.133
In-hospital mortality, n (%)	75 (4.4)	68 (0.8)	< 0.001

Data are presented as mean (SD) or n (%) as appropriate. ICU length of stay was calculated among patients admitted to the ICU

*ICU* intensive care unit, *SLE* systemic lupus erythematosus

In multivariable logistic regression restricted to patients with SLE (Table 5), cardiovascular risk was associated with clinical complications (adjusted OR 2.75, 95% CI 1.36–5.54; *p* = 0.004) and other procedure- or care-related complications (adjusted OR 5.46, 95% CI 1.84–16.18; *p* = 0.002). Cardiovascular risk was not significantly associated with surgical, infectious, or obstetric complications or with algorithm-defined SLE reactivation.

**Table 5:** Multivariable associations with postoperative outcomes among patients with SLE

Exposure	Outcome	Adjusted OR (95% CI)	p-value
Cardiovascular risk	Surgical complication	1.07 (0.62–1.84)	0.796
	Clinical complication	2.75 (1.36–5.54)	0.004
	Infectious complication	0.88 (0.36–2.18)	0.799
	Obstetric complication**	1.81 (0.57–5.78)	0.310
	Other procedure- or care-related complications*	5.46 (1.84–16.18)	0.002
	Algorithm-defined SLE reactivation	0.54 (0.17–1.73)	0.335
Multisystemic SLE	Surgical complication	0.93 (0.62–1.40)	0.725
	Clinical complication	0.61 (0.30–1.27)	0.211
	Infectious complication	0.98 (0.49–1.95)	0.954
	Obstetric complication**	0.74 (0.22–2.52)	0.608
	Other procedure- or care-related complications*	1.35 (0.38–4.77)	0.647
	Algorithm-defined SLE reactivation	4.60 (1.08–19.48)	0.039
Cutaneous lupus	Surgical complication	0.98 (0.61–1.56)	0.926
	Clinical complication	0.93 (0.46–1.88)	0.853
	Infectious complication	1.41 (0.72–2.78)	0.319
	Obstetric complication**	0.64 (0.21–1.96)	0.426
	Other procedure- or care-related complications*	0.45 (0.13–1.52)	0.220
	Algorithm-defined SLE reactivation	2.38 (1.23–4.61)	0.010
Lupus nephritis	Surgical complication	1.09 (0.69–1.73)	0.695
	Clinical complication	0.55 (0.22–1.38)	0.229
	Infectious complication	1.13 (0.48–2.68)	0.777
	Obstetric complication**	0.51 (0.14–1.84)	0.337
	Other procedure- or care-related complications*	0.93 (0.24–3.61)	0.910
	Algorithm-defined SLE reactivation	0.42 (0.12–1.47)	0.172

Separate multivariable logistic regression models were used to assess associations between cardiovascular risk and postoperative outcomes and between each SLE clinical subtype and postoperative outcomes. Each clinical subtype was analysed separately as a binary variable. Models were adjusted for age, sex, and multimorbidity

*CI* confidence interval, *OR* odds ratio, *SLE* systemic lupus erythematosus

*Other procedure- or care-related complications comprised events related to medical or surgical procedures or care that were not classified within the preceding complication categories

**Analyses of obstetric complications were restricted to patients undergoing an obstetric index procedure

Adjusted sensitivity analyses yielded largely consistent results (Table 6). After restriction to elective procedures, SLE was associated with increased risks of surgical, clinical, infectious, and other procedure- or care-related complications, whereas the association with obstetric complications was attenuated and was no longer statistically significant. After exclusion of patients classified with cutaneous lupus (L93), SLE remained associated with increased risks across all evaluated complication categories, including obstetric complications. In both sensitivity analyses, patients with SLE had a lower probability of an uncomplicated postoperative course.

**Table 6:** Sensitivity analyses of the adjusted association between systemic lupus erythematosus and postoperative outcomes

Outcome	Elective procedures only		Excluding cutaneous lupus codes (L93)	
	aRR (95% CI)	*p*-value	aRR (95% CI)	*p*-value
No postoperative complications*	0.79 (0.75–0.83)	< 0.001	0.83 (0.80–0.86)	< 0.001
Surgical complications	2.23 (1.78–2.77)	< 0.001	2.09 (1.77–2.46)	< 0.001
Clinical complications	2.56 (1.66–3.97)	< 0.001	2.55 (1.83–3.55)	< 0.001
Infectious complications	3.81 (2.57–5.66)	< 0.001	3.18 (2.41–4.21)	< 0.001
Obstetric complications	1.35 (0.50–3.68)	0.557	2.25 (1.50–3.38)	< 0.001
Other procedure-or care-related complications	15.56 (5.35–45.26)	< 0.001	12.26 (6.53–23.05)	< 0.001

Values are adjusted risk ratios with 95% confidence intervals comparing patients with SLE and matched controls. The first sensitivity analysis was restricted to elective procedures. The second excluded patients classified with cutaneous lupus codes (L93)

*‘No postoperative complications’ was defined as the absence of any of the evaluated postoperative complication categories during the 180-day follow-up. Models were adjusted for age, sex, preoperative surgical risk category, surgical category, clinical comorbidity, and cardiovascular risk, as applicable to each outcome

No statistically significant associations were detected between clinical subtypes and most postoperative complications in the adjusted analyses. Multisystemic SLE was associated with higher odds of algorithm-defined disease reactivation (adjusted OR 4.60, 95% CI 1.08–19.48; *p* = 0.039), and cutaneous lupus was also associated with higher odds of reactivation (adjusted OR 2.38, 95% CI 1.23–4.61; *p* = 0.010). Lupus nephritis showed no statistically significant association with algorithm-defined disease reactivation (adjusted OR 0.42, 95% CI 0.12–1.47; *p* = 0.172).

## Discussion

In this nationwide retrospective cohort study, patients with SLE undergoing abdominal, pelvic, urologic, or obstetric procedures had consistently higher frequencies of postoperative complications than matched controls. Differences encompassed surgical, clinical, infectious, obstetric, and other procedure- or care-related complications despite adequate post-matching balance. Within the SLE cohort, cardiovascular risk was independently associated with clinical and other procedure- or care-related complications.

These findings extend previous evidence from both population-based studies and smaller or procedure-specific cohorts [2–5, 12–15]. Prior studies have addressed perioperative immunosuppressive management in rheumatic diseases, early postoperative infection in relation to perioperative antirheumatic therapy, early outcomes after kidney transplantation in patients with SLE, and pregnancy outcomes in SLE populations [15–18]. The present study broadens this evidence by evaluating a nationwide matched cohort of patients with SLE undergoing a wide spectrum of surgical and obstetric procedures and by assessing multiple categories of postoperative complications within the same study.

Infectious and clinical complications were particularly increased among patients with SLE [5].

Other procedure- or care-related complications showed the largest relative difference between groups, although their absolute frequency was low. Cardiovascular risk was independently associated with these events and with clinical complications, highlighting the potential relevance of broader preoperative clinical risk assessment in SLE [4, 13, 14]. Obstetric complications were also more frequent among patients with SLE, consistent with the recognized complexity of pregnancy and obstetric care in this disease [19, 20].

Clinical heterogeneity was also evident. Patients with lupus nephritis had a higher baseline risk profile, including greater clinical comorbidity, cardiovascular risk, and a higher frequency of emergency inflammatory surgery, but lupus nephritis was not independently associated with the evaluated postoperative outcomes. The relatively small size of this subgroup (*n* = 202) may have limited statistical precision, and the nonsignificant estimate suggesting a lower likelihood of algorithm-defined SLE reactivation should not be interpreted as evidence of a protective effect. The administrative classification of cutaneous lupus also requires caution because patients classified with L93 may have included both severe or refractory cutaneous disease and incompletely coded systemic SLE. Importantly, exclusion of this subgroup did not materially alter the main associations with postoperative complications.

A major contribution of this study lies in its methodological approach. Linking AIH, BPA-I, and APAC records through an anonymised patient identifier enabled reconstruction of longitudinal healthcare trajectories across inpatient, outpatient, and high-complexity care within SUS. This integration allowed procedures, preoperative characteristics, treatment-related information, and postoperative events to be examined within a single analytical framework. More broadly, this approach illustrates how routinely collected data from a universal health system can generate population-level evidence in complex chronic diseases, particularly where prospective disease-specific cohorts are difficult to establish or sustain.

Clinical variables not captured in DATASUS, including measures of disease activity and treatment exposure, may have influenced postoperative risk estimates. Algorithm-defined SLE reactivation should therefore be interpreted as an administrative proxy rather than a clinically validated flare outcome. In addition, secondary subgroup findings, particularly those with borderline statistical significance, should be interpreted cautiously in the absence of formal adjustment for multiple comparisons.

The consistency of the main associations across the sensitivity analyses strengthens the robustness of the findings. Restriction to elective procedures reduced the potential influence of surgical urgency, whereas exclusion of patients classified with cutaneous lupus addressed possible diagnostic misclassification within the administrative dataset. In both analyses, the increased risks of surgical, clinical, and infectious complications associated with SLE were maintained, supporting the stability of the main results across alternative analytical definitions.

In conclusion, patients with SLE in this nationwide administrative cohort experienced higher rates of several postoperative complications compared with matched controls. These findings identify a higher-risk perioperative profile in this population, while the relative contributions of underlying disease activity and immunosuppressive treatment warrant further investigation.

## Supplementary Information

Below is the link to the electronic supplementary material.

Supplementary Material 1

## Data Availability

The data analysed in this study were obtained from DATASUS through an official request. Access to the underlying individual-level data is subject to DATASUS data-access procedures and applicable confidentiality requirements.

## Declarations

### Disclaimer

No part of this manuscript has been copied from or published elsewhere, and no scientific content, study data, statistical analyses, results, or conclusions were generated by artificial intelligence. The limited use of artificial intelligence for language editing, text organisation, and refinement of author-generated text is disclosed separately in the manuscript. The authors take full responsibility for the content and integrity of this report.
