# Supplementary material for: Higher postoperative complication rates in patients with systemic lupus erythematosus undergoing surgical and obstetric procedures: a nationwide retrospective matched cohort study
Source: Rheumatol Int. 2026 Oct 9;46(10):280. doi: 10.1007/s00296-026-06316-9 (PMC13652903; doi:10.1007/s00296-026-06316-9)
Supplement: Supplementary file 1 [file 296_2026_6316_MOESM1_ESM.docx]

*Higher Postoperative Complication Rates in Patients with Systemic Lupus Erythematosus Undergoing Surgical and Obstetric Procedures: A Nationwide Retrospective Matched Cohort Study*

**Authors:**

Francisco Eduardo Silva^1^
Mirhelen Mendes de Abreu^2^

1. **Francisco Eduardo Silva, MD, MSc**

E-mail: [franciscoeduardo@macae.ufrj.br](mailto:franciscoeduardo@macae.ufrj.br)

ORCID: 0000-0001-6553-4634

Universidade Federal do Rio de Janeiro, Campus Macaé, Rio de Janeiro, Brazil.

Rua Aluízio da Silva Gomes, 50. Granja dos Cavaleiros. Macaé, Rio de Janeiro

CEP: 27930-560

2. **Mirhelen Mendes de Abreu, MD, PhD (Corresponding author)**

E-mail: [mirhelen@hucff.ufrj.br](mailto:mirhelen@hucff.ufrj.br)

ORCID: 0000-0002-2264-0442

Universidade Federal do Rio de Janeiro, Campus Cidade Universitária, Rio de Janeiro, Brazil.

Rua Professor Rodolpho Paulo Rocco, 255, Rio de Janeiro, Rio de Janeiro

CEP: 21941-913

# Data Sources and Database Architecture

DATASUS (Departamento de Informática do Sistema Único de Saúde) is the information technology department of the Brazilian Unified Health System (Sistema Único de Saúde [SUS]). It maintains nationwide administrative healthcare databases generated primarily for reimbursement, healthcare management, and health-system monitoring. Because these data are collected for administrative rather than research purposes, they may be subject to coding variability and lack some granular clinical information.

Three complementary DATASUS databases were integrated in this study: the Hospital Admission Authorization database (Autorização de Internação Hospitalar [AIH]), the Authorization for High-Complexity Outpatient Procedures database (Autorização de Procedimentos de Alta Complexidade [APAC]), and the Individualized Outpatient Production Bulletin (Boletim de Produção Ambulatorial Individualizado [BPA-I]). Together, these sources provide information on inpatient admissions, diagnoses, surgical and obstetric procedures, outpatient care, and high-complexity treatment within SUS.

**Figure S1. Integration of DATASUS databases and construction of the longitudinal study cohort.**

# Patient-Level Linkage and Data Quality Procedures

Records from AIH, APAC, and BPA-I were linked using a unique anonymized patient identifier provided within the study dataset. This identifier enabled longitudinal tracking of healthcare records across inpatient and outpatient databases.

Data quality procedures included assessment of temporal consistency, duplicate identification, and validation of repeated records across databases. Records with chronologically inconsistent dates were excluded from analyses requiring temporal sequencing. Fully duplicated administrative records were collapsed into a single observation, whereas distinct procedures occurring on different dates were retained as separate healthcare events.

# Database-Specific Variables Used in the Study

The tables below retain variables directly relevant to cohort construction, linkage, exposures, matching, procedures, and outcomes. Administrative fields not used analytically were omitted.

## Table S1. AIH variables used in the study

| **Variable** | **Definition / analytical use** |
| --- | --- |
| ID_PACIENTE | Unique anonymized patient identifier used for linkage. |
| DT_INTERNAÇÃO | Hospital admission date. |
| DT_SAIDA | Hospital discharge date. |
| CO_PACIENTE_SEXO | Patient sex. |
| VL_PACIENTE_IDADE | Patient age. |
| NU_PACIENTE_MUN_ORIGEM/ geographic fields | Municipality/state information used to derive geographic region. |
| CO_CID_PRINCIPAL | Primary ICD-10 diagnosis code. |
| CO_CID_SECUNDARIO and secondary diagnosis fields | Secondary ICD-10 diagnosis codes. |
| CO_PROCEDIMENTO_PRINCIPAL | Main procedure performed. |
| COD_PROCEDIMENTO_SECUNDARIO | Secondary procedure code(s). |
| CO_CAR_INTERNAÇÃO | Character/type of hospitalization. |

## Table S2. APAC variables used in the study

| **Variable** | **Definition / analytical use** |
| --- | --- |
| ID PACIENTE | Unique anonymized patient identifier used for linkage. |
| DATA INICIO/ DATA FIM | Validity period of the APAC record. |
| CO_CID_PRINCIPAL | Primary ICD-10 diagnosis code. |
| CO_CID_SECUNDÁRIO | Secondary ICD-10 diagnosis code. |
| CO_PROCEDIMENTO_PRINCIPAL | Primary outpatient procedure code. |
| CO_PROCEDIMENTO_SECUNDARIO | Secondary outpatient procedure code(s). |
| QT_PROCEDIMENTO | Number of procedures recorded. |
| DATA SOLICITAÇÃO | Claim/request date. |
| DATA GERAÇÃO | Approval/processing date. |
| DATA OCORRÊNCIA | Recorded occurrence/completion date. |
| IDADE/ SEXO | Age and sex. |
| UF_ENDEREÇO/ municipality fields | Geographic information. |

## Table S3. BPA-I variables used in the study

| **Variable** | **Definition / analytical use** |
| --- | --- |
| ID_paciente | Unique anonymized patient identifier used for linkage. |
| dt_atendimento | Date of outpatient service. |
| co_procedimento_realizado | Procedure code. |
| co_cid_principal | Primary ICD-10 diagnosis code. |
| co_carater_atendimento | Service characteristic. |
| dt_nascimento / idade | Birth date/age. |
| sexo | Sex. |
| UF_paciente / municipio_paciente | Geographic information. |
| CNES | Health establishment code. |

# SLE Case Definition and Clinical Subtype Classification

Patients were identified using a predefined administrative algorithm combining diagnostic and treatment records. Eligible individuals were required to have ICD-10 codes compatible with systemic lupus erythematosus or lupus-related disease (M32.x, L93.x, or N08.x), together with outpatient treatment records compatible with SLE management according to the Brazilian Clinical Protocol and Therapeutic Guidelines in effect during the study period. Eligibility additionally required an abdominal, pelvic, urologic, or obstetric procedure recorded in AIH and documented immunosuppressive treatment during the 12 months preceding the index procedure.

Patients were classified into mutually exclusive administrative clinical subtypes using predefined diagnostic and treatment criteria: multisystemic SLE, cutaneous lupus, and lupus nephritis. Classification required at least two records of the same subtype-specific ICD-10 code within a 6-month window before or after the index procedure, together with at least two corresponding procedure records defined by the 2013 Brazilian Clinical Protocol and Therapeutic Guidelines for systemic lupus erythematosus.

## Table S4. Administrative algorithm for SLE case identification and subtype classification

| **Component** | **Operational criterion** |
| --- | --- |
| SLE/lupus-related diagnostic record | ICD-10 M32.x, L93.x, or N08.x. |
| Treatment criterion | Outpatient treatment record compatible with SLE management under the Brazilian Clinical Protocol and Therapeutic Guidelines in effect during the study period. |
| Index procedure criterion | Abdominal, pelvic, urologic, or obstetric procedure recorded in AIH. |
| Preoperative treatment criterion | Documented immunosuppressive treatment during the 12 months preceding the index procedure. |
| Clinical subtype | Mutually exclusive classification as multisystemic SLE, cutaneous lupus, or lupus nephritis, requiring at least two records of the same subtype-specific ICD-10 code within a 6-month window before or after the index procedure, together with at least two corresponding procedure records defined by the 2013 Brazilian Clinical Protocol and Therapeutic Guidelines for SLE. |

# Control Selection and Matching Strategy

Controls were selected among individuals undergoing the same categories of abdominal, pelvic, urologic, or obstetric procedures who had no recorded diagnosis of SLE or other autoimmune disease and no condition associated with secondary immunosuppression.

Each patient with SLE was matched to five controls according to sex, age group, geographic macro-region, preoperative surgical risk category, and surgical category. When more than five eligible controls were available within the same matching stratum, the first five eligible records were selected according to record availability. Post-matching balance was evaluated using standardized mean differences. Individuals with malignant neoplasms, other autoimmune diseases requiring immunosuppressive therapy, AIDS, or other conditions associated with secondary immunosuppression were excluded.

# Surgical Identification and Preoperative Risk Variables

Surgical procedures were identified using SUS procedure codes obtained from the SIGTAP (Sistema de Gerenciamento da Tabela de Procedimentos) classification system. Procedure descriptions were retained according to official SIGTAP terminology to support reproducibility.

**Table S5. SUS/SIGTAP procedure codes screened in the administrative databases for identification of eligible surgical and obstetric procedures**

| **SUS/SIGTAP code** | **Procedure description** |
| --- | --- |
| 04.07.01.001-7 | degastrogastrectomy with or without vagotomy |
| 04.07.01.002-5 | esophageal/pyloric dilation |
| 04.07.01.003-3 | distal esophagectomy with thoracotomy |
| 04.07.01.004-1 | distal esophagectomy without thoracotomy |
| 04.07.01.005-0 | videolaparoscopic esophagectomy |
| 04.07.01.006-8 | esophagocolonplasty |
| 04.07.01.007-6 | esophagogastrectomy |
| 04.07.01.008-4 | esophagoplasty/gastroplasty |
| 04.07.01.009-2 | cervical esophagorrhaphy |
| 04.07.01.010-6 | thoracic esophagorrhaphy |
| 04.07.01.011-4 | esophagostomy |
| 04.07.01.012-2 | gastrectomy with or without duodenal diversion |
| 04.07.01.013-0 | partial gastrectomy with or without vagotomy |
| 04.07.01.014-9 | total gastrectomy |
| 04.07.01.015-7 | videolaparoscopic gastrectomy |
| 04.07.01.016-5 | gastroenteroanastomosis |
| 04.07.01.017-3 | gastroplasty with intestinal diversion |
| 04.07.01.018-1 | vertical banded gastroplasty |
| 04.07.01.019-0 | gastrorrhaphy |
| 04.07.01.020-3 | videolaparoscopic gastrorrhaphy |
| 04.07.01.021-1 | gastrostomy |
| 04.07.01.022-0 | videolaparoscopic gastrostomy |
| 04.07.01.023-8 | pyloroplasty |
| 04.07.01.024-6 | removal of foreign body from the GI tract by endoscopy |
| 04.07.01.025-4 | removal of polyp from the digestive tract by endoscopy |
| 04.07.01.026-2 | tamponade of hemorrhagic lesions of the GI tract |
| 04.07.01.027-0 | surgical treatment of achalasia (cardiomyoplasty) |
| 04.07.01.028-9 | treatment surgical treatment of diverticulum of the digestive tract |
| 04.07.01.029-7 | surgical treatment of gastroesophageal reflux |
| 04.07.01.030-0 | surgical treatment of esophageal varices |
| 04.07.01.031-9 | sclerosing treatment/tgi rubber band ligation |
| 04.07.01.032-7 | sclerosing treatment of non-hemorrhagic lesions |
| 04.07.01.033-5 | surgical treatment of megaesophagus without resection |
| 04.07.01.034-3 | vagotomy with drainage operation |
| 04.07.01.035-1 | superselective vagotomy/proximal gastric |
| 04.07.02.002-0 | amputation due to prolapse of rectum |
| 04.07.02.003-9 | appendectomy |
| 04.07.02.004-7 | videolaparoscopic appendectomy |
| 04.07.02.005-5 | cerclage of the anus |
| 04.07.02.006-3 | partial colectomy (hemicolectomy) |
| 04.07.02.007-1 | total colectomy |
| 04.07.02.008-0 | videolaparoscopic colectomy |
| 04.07.02.009-8 | abdominal colorrhaphy |
| 04.07.02.010-1 | colostomy |
| 04.07.02.011-0 | single/multiple cryptectomy |
| 04.07.02.012-8 | digital/instrumental dilation of the anus and/or rectum |
| 04.07.02.013-6 | drainage of anurethral abscess |
| 04.07.02.014-4 | drainage of ischiorectal abscess |
| 04.07.02.015-2 | drainage of retrorectal hematoma/abscess |
| 04.07.02.016-0 | electrocautery of transparietal lesion of anus |
| 04.07.02.017-9 | enterectomy |
| 04.07.02.018-7 | enteroanastomosis (any segment) |
| 04.07.02.019-5 | enteropexy (any segment) |
| 04.07.02.020-9 | enterotomy and/or enterorrhaphy with suture/resection |
| 04.07.02.021-7 | internal sphincterotomy and treatment of anal fissure |
| 04.07.02.022-5 | excision of anurethral lesion/tumor |
| 04.07.02.023-3 | excision of localized intestinal/mesenteric lesion |
| 04.07.02.024-1 | closure of enterostomy (any segment) |
| 04.07.02.025-0 | closure of colonic fistula |
| 04.07.02.026-8 | closure of rectal fistula |
| 04.07.02.027-6 | fistulectomy/anal fistulotomy |
| 04.07.02.028-4 | hemorrhoidectomy |
| 04.07.02.029-2 | herniorrhaphy with intestinal resection |
| 04.07.02.030-6 | jejunostomy/ileostomy |
| 04.07.02.032-2 | external anal plastic surgery / anal sphincteroplasty |
| 04.07.02.033-0 | total proctocolectomy with ileal reservoir |
| 04.07.02.034-9 | abdominal proctopexy due to prolapse of the rectum |
| 04.07.02.035-7 | proctoplasty and proctorrhaphy via the perineal route |
| 04.07.02.036-5 | surgical reduction of volvulus by laparotomy |
| 04.07.02.039-0 | removal of foreign body |
| 04.07.02.040-3 | abdominal rectosigmoidectomy |
| 04.07.02.041-1 | abdominoperineal rectosigmoidectomy |
| 04.07.02.047-0 | surgical treatment of anal prolapse |
| 04.07.02.048-9 | surgical treatment of anal pruritus |
| 04.07.03.001-8 | bileodigestive anastomosis |
| 04.07.03.002-6 | cholecystectomy |
| 04.07.03.003-4 | videolaparoscopic cholecystectomy |
| 04.07.03.004-2 | cholecystostomy |
| 04.07.03.005-0 | choledochoplasty |
| 04.07.03.006-9 | choledochotomy with or without cholecystectomy |
| 04.07.03.007-7 | videolaparoscopic choledochotomy |
| 04.07.03.008-5 | biliary stent placement |
| 04.07.03.009-3 | dilatation percutaneous biliary stricture repair |
| 04.07.03.010-7 | external percutaneous biliary drainage |
| 04.07.03.011-5 | internal percutaneous biliary drainage |
| 04.07.03.012-3 | splenectomy |
| 04.07.03.013-1 | partial hepatectomy |
| 04.07.03.014-0 | hepatorrhaphy |
| 04.07.03.015-8 | complex hepatorrhaphy |
| 04.07.03.016-6 | hepatotomy and abscess/cyst drainage |
| 04.07.03.017-4 | abscess/cyst marsupialization |
| 04.07.03.018-2 | partial pancreatectomy |
| 04.07.03.019-0 | pancreatectomy videolaparoscopic |
| 04.07.03.020-4 | pancreatoduodenectomy |
| 04.07.03.021-2 | pancreatoenterostomy |
| 04.07.03.022-0 | pancreatotomy for drainage |
| 04.07.03.023-9 | percutaneous removal of gallstones |
| 04.07.03.024-7 | surgical treatment of pancreatic cysts |
| 04.07. 04.001-3 | pelvic abscess drainage |
| 04.07.04.002-1 | subphrenic abscess drainage |
| 04.07.04.003-0 | preperitoneal hematoma/abscess drainage |
| 04.07.04.004-8 | diaphragmatic hernioplasty (abdominal approach) |
| 04.07.04.005-6 | diaphragmatic hernioplasty (thoracic approach) |
| 04.07.04.006-4 | epigastric hernioplasty |
| 04.07.04.007-2 | videolaparoscopic epigastric hernioplasty |
| 04.07.04.008-0 | incisional hernioplasty |
| 04.07.04.009-9 | inguinal hernioplasty (bilateral) |
| 04.07.04.010-2 | inguinal/crural hernioplasty (unilateral) |
| 04.07.04.011-0 | recurrent hernioplasty |
| 04.07.04.012-9 | umbilical hernioplasty |
| 04.07.04.013-7 | videolaparoscopic inguinal herniorrhaphy |
| 04.07.04.014-5 | herniorrhaphy without intestinal resection |
| 04.07.04.015-3 | videolaparoscopic umbilical herniorrhaphy |
| 04.07.04.016-1 | exploratory laparotomy |
| 04.07.04.017-0 | videolaparoscopic laparotomy |
| 04.07.04.018-8 | release of intestinal adhesions |
| 04.07.04.019-6 | abdominal paracentesis |
| 04.07.04.020-0 | peritoniostomy with inorganic mesh |
| 04.07.04.021-8 | pneumoperitoneum (per session) |
| 04.07.04.022-6 | repair of other hernias |
| 04.07.04.023-4 | epiplum resection |
| 04.07.04.024-2 | abdominal wall resuturing |
| 04.07.04.025-0 | surgical treatment of peritonitis |
| 04.07.04.026-9 | videolaparoscopic vagotomy |
| 04.09.06.013-5 | total hysterectomy |
| 04.09.06.014-3 | extended total hysterectomy (werthein-meigs) |
| 04.09.06.015-1 | videolaparoscopic hysterectomy |
| 04.09.06.016-0 | hysterorrhaphy |
| 04.09.06.017-8 | surgical hysteroscopy with resectoscope |
| 04.09.06.018-6 | tubal ligation |
| 04.09.06.019-4 | myomectomy |
| 04.09.06.020-8 | videolaparoscopic myomectomy |
| 04.09.06.021-6 | oophorectomy / oophoroplasty |
| 04.09.06.022-4 | pelvic varicose vein resection |
| 04.09.06.023-2 | uni / bilateral salpingectomy |
| 04.09.06.024-0 | salpingectomy videolaparoscopic |
| 04.09.06.025-9 | salpingoplasty |
| 04.09.06.026-7 | videolaparoscopic salpingoplasty |
| 04.09.06.028-3 | surgical treatment of vesicouterine fistula |
| 04.09.06.029-1 | hysterectomy with bilateral adnexectomy |
| 04.09.07.002-5 | colpectomy |
| 04.09.07.003-3 | colpocleisis (le fort surgery) |
| 04.09.07.004-1 | colpoperineocleisis |
| 04.09.07.005-0 | anterior and posterior colpoperineoplasty |
| 04.09.07.006-8 | posterior colpoperineoplasty |
| 04.09.07.007-6 | non-obstetric colpoperineorrhaphy |
| 04.09.07.008-4 | anterior colpoplasty |
| 04.09.07.009-2 | non-obstetric colporrhaphy |
| 04.09.07.010-6 | colpotomy |
| 04.09.07.011-4 | construction of vagina |
| 04.09.07.012-2 | drainage of bartholin/skene gland |
| 04.09.07.013-0 | non-obstetric episioperineorrhaphy |
| 04.09.07.014-9 | excision of vaginal cyst |
| 04.09.07.015-7 | excision of bartholin/skene gland |
| 04.09.07.016-5 | excision of vulvar lesion |
| 04.09.07.017-3 | extraction of foreign body from the vagina |
| 04.09.07.018-1 | hymenotomy |
| 04.09.07.019-0 | marsupialization of bartholin's gland |
| 04.09.07.020-3 | burch operation |
| 04.09.07.021-1 | reconstruction of the vagina |
| 04.09.07.022-0 | surgical treatment of coaptation of nymphs |
| 04.09.07.023-8 | surgical treatment of rectovaginal fistula |
| 04.09.07.024-6 | surgical treatment of urethrovaginal fistula |
| 04.09.07.025-4 | surgical treatment of vesicovaginal fistula |
| 04.09.07.026-2 | surgical treatment of hypertrophy of the labia minora |
| 04.09.07.027-0 | surgical treatment of urinary incontinence |
| 04.09.07.028-9 | surgical treatment of septate/atretic vagina |
| 04.09.07.031-9 | female sex reassignment |
| 04.11.01.001-8 | manual placental abruption |
| 04.11.01.002-6 | cesarean section in high-risk pregnancy |
| 04.11.01.003-4 | cesarean section |
| 04.11.01.004-2 | cesarean section with tubal ligation |
| 04.11.01.005-0 | manual reduction of acute postpartum uterine inversion |
| 04.11.01.006-9 | postpartum episiorrhaphy re-suturing |
| 04.11.01.007-7 | suturing of pelvic tract lacerations |
| 04.11.01.008-5 | surgical treatment of acute uterine inversion |
| 04.11.02.001-3 | post-abortion/puerperal curettage |
| 04.11.02.002-1 | embryotomy |
| 04.11.02.003-0 | puerperal hysterectomy |
| 04.11.02.004-8 | surgical treatment of ectopic pregnancy |
| 04.13.04.004-6 | non-aesthetic abdominal dermolipectomy |
| 04.13.04.005-4 | post-bariatric surgery abdominal dermolipectomy |
| 04.13.04.006-2 | brachial dermolipectomy after bariatric surgery |
| 04.13.04.007-0 | crural dermolipectomy after bariatric surgery |
| 04.06.01.012-9 | intra-aortic balloon pump placement |
| 04.06.01.013-7 | aneurysm repair |
| 04.06.01.124-9 | coarctation of the aorta with ecs |
| 04.06.02.001-9 | splenorenal anastomosis / other central shunt |
| 04.06.02.002-7 | lymphovenous anastomosis |
| 04.06.02.003-5 | portocaval anastomosis |
| 04.06.02.004-3 | infrarenal abdominal aortic aneurysmectomy |
| 04.06.02.005-1 | thoracoabdominal aneurysmectomy |
| 04.06.02.054-0 | surgical treatment of vascular lesions of the abdomen |
| 04.06.02.058-2 | replacement of descending aorta (includes abdominal) |
| 04.06.02.059-0 | thrombectomy of the venous system |
| 04.06.04.014-1 | percutaneous vena cava filter placement |
| 04.06.04.015-0 | endovascular aneurysm repair |
| 04.06.04.016-8 | endovascular aneurysm repair |
| 04.06.04.017-6 | endovascular aneurysm repair |
| 04.06.04.018-4 | endovascular aneurysm repair |
| 04.06.04.019-2 | arterial embolization of gastrointestinal bleeding |
| 0406020019 | splenorenal anastomosis/other central bypass |
| 0406020027 | lymphovenous anastomosis |
| 0406020035 | portocaval anastomosis |
| 0406020043 | infrarenal abdominal aortic aneurysmectomy |
| 0406020051 | aneurysmectomy thoraco-abdominal |
| 0409010189 | lithotripsy |
| 0409010197 | lumbotomy |
| 0409010200 | partial nephrectomy |
| 0409010219 | total nephrectomy |
| 0409010227 | nephrolithotomy |
| 0409010235 | percutaneous nephrolithotomy |
| 0409010243 | nephropexy |
| 0409010251 | nephropyelostomy |
| 0409010260 | nephrorrhaphy |
| 0409010278 | nephrostomy (by puncture |
| 0409010294 | percutaneous nephrostomy |
| 0409010308 | total nephroureterectomy |
| 0409010316 | pyelolithotomy |
| 0409010324 | pyeloplasty |
| 0409010332 | pyelostomy |
| 0409010340 | pyelotomy |
| 0409010413 | surgical treatment of neurogenic bladder |
| 0409010421 | surgical treatment of renal cyst by puncture |
| 0409010430 | surgical treatment of cystocele |
| 0409010448 | surgical treatment of vesicocutaneous fistula |
| 0409010456 | surgical treatment of vesicoenteric fistula |
| 0409010464 | surgical treatment of vesicorectal fistula |
| 0409010472 | surgical treatment of ureteral fistulas |
| 0409010480 | surgical treatment of bladder hemorrhage |
| 0409010499 | surgical treatment of urinary incontinence |
| 0409010502 | surgical treatment of vesicoureteral reflux |
| 0409010510 | surgical treatment of ureterocele |
| 0409010529 | ureterectomy |
| 0409010537 | ureterocystoneostomy |
| 0409010545 | ureteroenteroplasty |
| 0409010553 | ureteroenterostomy |
| 0409010561 | ureterolithotomy |
| 0409010570 | ureteroplasty |
| 0409010588 | cutaneous ureterostomy |
| 0409020010 | drainage of periurethral collection |
| 0409020028 | drainage of urinary phlegmon |
| 0409020036 | endoscopic extraction of foreign body |
| 0409010014 | capsulectomy renal |
| 0409010057 | cystoenteroplasty |
| 0409010065 | cystolithotomy and/or removal of foreign body from the bladder |
| 0409010073 | cystoplasty (correction of bladder exstrophy) |
| 0409010081 | cystorrhaphy |
| 0409010090 | cystostomy |
| 0409020168 | perineal/cutaneous/external urethrostomy |
| 0409020176 | internal urethrotomy |
| 0409020184 | urethrotomy to remove stone or foreign body |
| 0409030015 | drainage of prostatic abscess |
| 0409030023 | suprapubic prostatectomy |
| 0409030031 | radical prostatovesiculectomy |
| 0409030040 | endoscopic resection of prostate |

## Preoperative risk variables

Preoperative risk characterization was based on the 3rd Brazilian Society of Cardiology Guideline for Perioperative Cardiovascular Evaluation (2017). Because DATASUS does not provide granular physiological or laboratory measurements, risk domains were operationalized using diagnostic and procedure coding proxies available in AIH, APAC, and BPA-I.

## Table S6. Operational definitions of preoperative risk variables

| **Domain** | **Operational definition** |
| --- | --- |
| Cardiovascular risk | ICD-10 codes corresponding to hypertension, ischemic heart disease, heart failure, arrhythmias, valvular heart disease, conduction disorders, and cerebrovascular disease. |
| Clinical comorbidity | Conditions identified according to the study's operationalization of the SBC guideline, including diabetes mellitus, renal insufficiency, pulmonary disease, adrenal disorders, anemia, thyroid disease, obesity, and smoking-related conditions. |
| Multimorbidity | Clinical multimorbidity was operationalized as the presence of two or more selected chronic conditions recorded during the preoperative period and included as a factor potentially influencing surgical outcomes. |
| Obstetric risk | Pregnancy-related conditions identified using ICD-10 codes reflecting gestational care and pregnancy-related complications relevant to procedural outcomes. |

# Operational Definitions of Postoperative Outcomes

Five categories of postoperative complications were evaluated: surgical complications, including reoperation, abscess, wound dehiscence, and hemorrhagic events; clinical complications, including organ failure and cardiovascular or respiratory events; infectious complications, including sepsis and surgical-site, urinary, or respiratory infections; obstetric complications, including miscarriage, hemorrhage, eclampsia, and preeclampsia; and other procedure- or care-related complications not classified within the preceding categories.

The latter category was identified using ICD-10 codes related to complications of medical or surgical procedures and care, including T81.x, T88.x, Y60.x, Y61, Y65.2, Y83, and K91.4.

Postoperative complications were recorded during the 180-day follow-up after the index procedure. Outcome ascertainment was performed using linked administrative records across the available DATASUS databases.

Postoperative complications were identified using diagnostic and procedure records during the 180 days following the index procedure. Outcomes included surgical, clinical, infectious, obstetric, and other procedure- or care-related complications, as well as algorithm-defined SLE reactivation. Outcome categories were analyzed independently, allowing patients to contribute to more than one complication category when clinically plausible. Postoperative SLE reactivation was defined using an administrative algorithm based on a new record compatible with active SLE together with resumption or escalation of immunosuppressive treatment.

**Methodological Considerations**

DATASUS databases are administrative data sources originally designed for healthcare management and reimbursement rather than clinical research. Accordingly, diagnostic and procedural misclassification may occur, and granular clinical measures such as validated disease activity scores, laboratory parameters, physiological measurements, and detailed treatment information are not consistently available. These limitations were addressed, when possible, through predefined coding algorithms, cross-database validation, and longitudinal linkage of complementary data sources.

Medication-related records were available through the administrative databases; however, adherence, exact administered dose, and all clinical indications could not be fully ascertained from administrative records.

**References**

1. Ministry of Health (Brazil) (2013) Clinical Protocol and Therapeutical Guidelines – systemic lupus erythematosus. Ministry of Health, Brasília (DF). Portaria SAS/MS nº 100, February 7, 2013. Diário Oficial da União 2013 Feb 8; Sec 1:70–75. <https://www.gov.br/conitec/pt-br/midias/protocolos/lupuseritematoso_sistemico-1.pdf>. Accessed March 31, 2023
2. Ministry of Health (Brazil). 2022 Clinical Protocol and Therapeutical Guidelines – systemic lupus erythematosus. Brasília (DF): Ministry of Health; 2022. Portaria SCTIE/MS nº 43, September 28, 2022. Diário Oficial da União 2022 Sep 29; Sec 1:123–6. [https://www.gov.br/conitec/pt-br/midias/protocolos/pcdt_lupus_eritematoso_sistemico_2022.pdf.](https://www.gov.br/conitec/pt-br/midias/relatorios/portaria/2022/20221109_portaria_conjunta_n21.pdf) Accessed March 31, 2023.
3. Gualandro DM, Yu PC, Caramelli B, Marques AC, Calderaro D, Fornari LS, Pinho C, Feitosa ACR, Polanczyk CA, Rochitte CE, Jardim C, Vieira CLZ, Nakamura DYM, Iezzi D, Schreen D, Adam EL, D'Amico EA, Lima EQ, Burdmann EA, Mateo EIP et al (2017) 3rd Guideline for Perioperative Cardiovascular Evaluation of the Brazilian Society of Cardiology. Arq Bras Cardiol 109(3 Suppl 1):1–104. <https://doi.org/10.5935/abc.20170140>
